# Maternal colonization with *Staphylococcus aureus* and Group B streptococcus is associated with colonization in newborns

**DOI:** 10.1016/j.cmi.2017.04.020

**Published:** 2017-12

**Authors:** A. Roca, A. Bojang, B. Camara, C. Oluwalana, K. Lette, P. West, U. D'Alessandro, C. Bottomley

**Affiliations:** 1) Medical Research Council Unit The Gambia, Banjul, Gambia; 2) Faculty of Epidemiology and Population Health, London School of Hygiene & Tropical Medicine, London, UK; 3) Faculty of Infectious and Tropical Diseases, London School of Hygiene & Tropical Medicine, London, UK; 4) Institute of Tropical Medicine, Antwerp, Belgium

**Keywords:** Africa, Colonization, Group B streptococcus, Mother–child, Risk factors, *Staphylococcus aureus*, Vertical transmission

## Abstract

**Objectives:**

Although *Staphylococcus aureus* and Group B streptococcus (GBS) are major causes of neonatal sepsis in sub-Saharan Africa, it is unclear how these bacteria are transmitted to the neonate.

**Methods:**

In a cohort of 377 Gambian women and their newborns, nasopharyngeal swabs were collected at delivery (day 0), and 3, 6, 14 and 28 days later. Breast milk samples and vaginal swabs were collected from the mother. *Staphylococcus aureus* and GBS were isolated using conventional microbiological methods.

**Results:**

Most women were carriers of *S. aureus* (264 out of 361 with all samples collected, 73.1%) at some point during follow up and many were carriers of GBS (114 out of 361, 31.6%). Carriage of *S. aureus* was common in all three maternal sites and GBS was common in the vaginal tract and breast milk. Among newborns, carriage of *S. aureus* peaked at day 6 (238 out of 377, 63.1%) and GBS at day 3 (39 out of 377, 10.3%). Neonatal carriage of *S. aureus* at day 6 was associated with maternal carriage in the breast milk adjusted OR 2.54; 95% CI 1.45–4.45, vaginal tract (aOR 2.55; 95% CI 1.32–4.92) and nasopharynx (aOR 2.49; 95% CI 1.56–3.97). Neonatal carriage of GBS at day 6 was associated with maternal carriage in the breast milk (aOR 3.75; 95% CI 1.32–10.65) and vaginal tract (aOR 3.42; 95% CI 1.27–9.22).

**Conclusions:**

Maternal colonization with *S. aureus* or GBS is a risk factor for bacterial colonization in newborns.

## Introduction

Among 6 million deaths in the under-fives occurred in 2013, more than half died of infections and approximately 44% occurred during the neonatal period [1]. In sub-Saharan Africa, *Staphylococcus aureus* [1] and Group B streptococcus (GBS) are common causes of severe bacterial disease in neonates. However, few studies have investigated how neonates become colonized—a necessary step to disease—with these bacteria.

In sub-Saharan Africa *S. aureus* colonization peaks during the first week of life (c.80% prevalence), and then decreases steadily until reaching a low plateau (c.20% prevalence) 10–20 weeks later [2], [3]. The timing of the peak during the neonatal period probably reflects the role of vertical transmission. Indeed, studies in developed countries have shown that vaginal colonization in the mother and breastfeeding are risk factors for *S. aureus* colonization during the neonatal period [4], [5], [6]. One study conducted in sub-Saharan Africa showed that mammillary colonization was a risk factor for *S. aureus* carriage during early infancy [7].

Rectovaginal GBS colonization is common during pregnancy, varying by geographical region from 11.1% in southern Asia to 22.4% in sub-Saharan Africa [8], and is a major risk factor for neonatal carriage [9], [10], [11]. It is estimated that 30%–70% of neonates born to GBS-colonized mothers are colonized by bacteria from the mother, and 1%–3% of those colonized develop severe disease.

Previous studies have shown that maternal and neonatal *S. aureus* and GBS colonization in The Gambia is high [3], [11], [12]. In this cohort study, we investigate the relationship between maternal colonization at various body sites—nasopharynx, vagina and breast milk—and nasopharyngeal *S. aureus* and GBS colonization in Gambian newborns.

## Material and methods

### Study design

This cohort study was a secondary analysis of data from the placebo arm of a double-blind, placebo-controlled randomized trial in which women in labour were randomized to receive a single dose of 2 g of oral azithromycin or placebo (ratio 1: 1) [13]. Women were recruited into the trial between April 2013 and April 2014 when attending the study health facility during labour; they had provided written informed consent to participate in the study during previous antenatal care visits. Women and newborns were followed for up to 8 weeks postpartum and biological samples were collected during the first 4 weeks [12], [14].

### Study site

The study was conducted at the Jammeh Foundation for Peace, a government-run health centre located in Western Gambia that manages 4500 deliveries/year. The population covers the main ethnic groups in The Gambia and illiteracy is high. The climate of the area is typical of the sub-Sahel region [15].

### Study samples

During labour a nasopharyngeal swab and a vaginal swab were collected from each woman, and within 4 h of birth a nasopharyngeal swab was collected from the newborn (day 0). Additional breast milk samples and maternal and newborn nasopharyngeal swabs were collected at days 3, 6, 14 and 28 during household visits conducted by study nurses and field workers. A second vaginal swab was collected by a study nurse or clinician when the mother visited the health facility between day 8 and day 13. Sample collection was stopped if the participant received antibiotics as part of standard care.

### Sample collection

Nasopharyngeal swabs were collected by inserting a calcium alginate (Expotech USA Inc., Houston, TX, USA) swab into the posterior wall of the nasopharynx and putting them into skimmed-milk–tryptone–glucose–glycerol as previously described [16]. Low vaginal swabs were collected by inserting a sterile cotton swab (Sterilin Ltd, Newport, UK) 2–3 cm into the vagina and rotating with a circular motion for 5 seconds, then placing the swabs into the vials containing skimmed-milk–tryptone–glucose–glycerol and placing in a cold box. To collect breast milk samples, the nipple and areola of the breast from which milk was taken was disinfected using sterile cotton soaked with 0.02% chlorhexidine. Mothers were then asked to manually express breast milk. The first 0.5 mL was discarded, and the following 1–2 mL was collected in another sterile plastic bijoux (Thermo Fisher Scientific, Loughborough, UK) bottle and put in a cold box. Samples were sent to the laboratory and arrived within 8 h, where they were vortexed and stored at –70°C.

### Laboratory procedures

Samples were thawed and processed in batches following standard procedures [13]. During processing, 50 μL of each vial was dispensed onto mannitol salt agar (CM0085; Oxoid, Basingstoke, UK) and crystal violet blood agar (CM0331; Oxoid, Basingstoke, UK +0.002% crystal violet) for selective isolation of *S. aureus* and GBS, respectively. After processing, samples were vortexed and then stored at –70°C.

### Data management, statistical analysis and study definitions

Case-report forms and laboratory forms were reviewed before being double entered into an *OpenClinica* (www.openclinica.com) database. Consistency checks and data validation were carried out regularly. All analyses were conducted using Stata 14.1. For each bacterium (*S. aureus* and GBS), we compared the prevalence of nasopharyngeal colonization at day 6 in neonates whose mothers were colonized at day 6 or earlier with the prevalence in neonates whose mothers were not colonized up to this time. Logistic regression was used to adjust for confounding due to maternal age and season. The population-attributable fraction—i.e. the proportion of colonization events attributable to maternal colonization—was calculated from the regression model using the ‘punaf’ command in Stata. We conducted separate analyses for the different maternal sources of bacteria (vaginal tract, nasopharynx and breast milk).

### Ethical clearance

The trial was approved by the joint Gambia Government/MRC Ethics Committees. An independent Data Safety and Monitoring Board monitored the data quality and treatment safety.

## Results

### Study participants

There were 424 children in the placebo arm of the trial. We excluded 18 twins, 27 children who were missing carriage data at day 6 and two children whose mothers were missing carriage data up to day 6. Hence the analysis was restricted to 377 newborns and their mothers. The characteristics of newborns and mothers included in this analysis are shown in Table 1. Overall, 98.7% (5954 out of 6032) of required samples were collected and included in the analysis.

**Table 1:** Baseline characteristics of study mothers and newborns

Characteristics		*n* (%)
*Mothers*		*n* = 377
Age, years, median (interquartile range)		25.0 (22.0–29.0)
Ethnicity^a^	Madinka	169 (45.4)
	Fula	57 (15.3)
	Jola	52 (14.0)
	Other	94 (25.3)
Season of delivery^b^	Dry	251 (66.6)
	Rainy	126 (33.4)
Mode of delivery	Vaginal	377 (100.0)
Time from rupture of membranes	≤18 h	351 (93.1)
to delivery	>18 h	26 (6.9)
*Newborns*		*n* = 377
Gender	Male	200 (53.1)
	Female	177 (46.9)
Apgar score^c^	7–10	372 (98.9)
	1–6	4 (1.1)
Birthweight^d^	(median, IQR)	3.1 (2.9–3.4)

a Ethnicity missing for *n* = 5 women.

b Rainy season: children born June to October.

c Apgar score missing in *n* = 1.

d Birthweight missing in *n* = 2.

### Maternal carriage of *S. aureus* and GBS

**Fig. 1:**
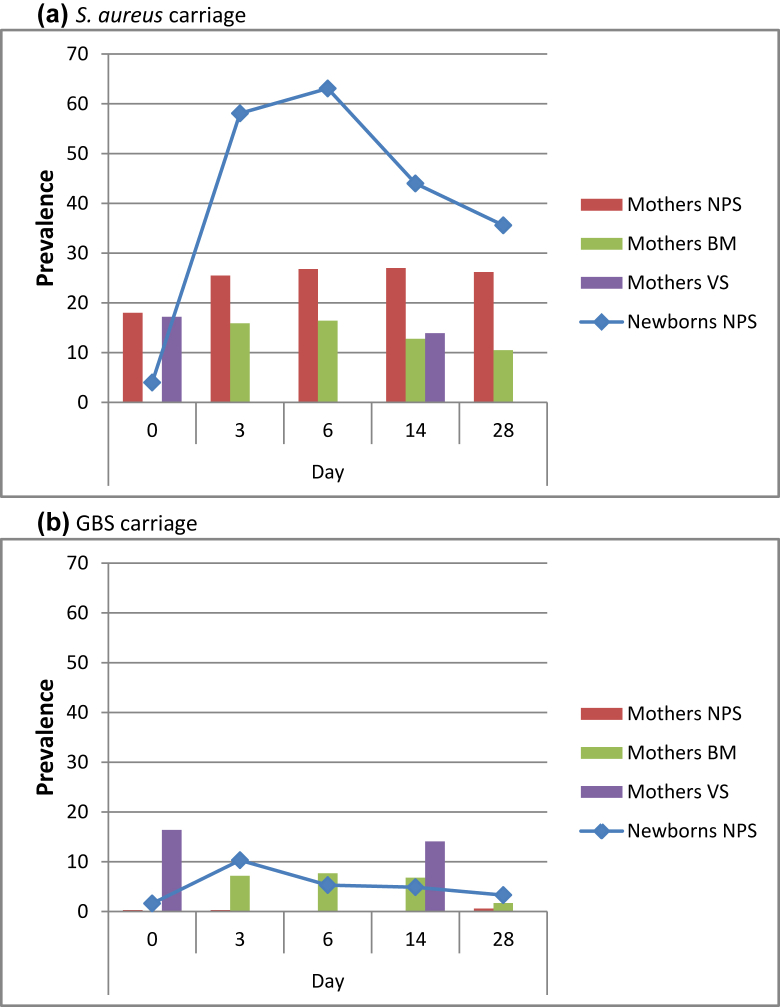
Prevalence (%) of maternal and neonatal carriage of (a) *Staphylococcus aureus* and (b) GBS during the follow-up period in the different biological sites (i.e. nasopharyngeal samples (NPS), breast milk samples (BM) and vaginal swabs (VS)). VS collected at day 8–13 were included in the table as Day 14.

Maternal *S. aureus* carriage during the 28 days following delivery is shown in Fig. 1(a). Most women (73.1%; 264 out of the 361 women with all the samples collected) were carriers of *S. aureus* at one or more time-points during the 28 days following delivery. Carriage at at least at one time-point was higher in nasopharyngeal swabs (51.5%; 186 out of 361 women with all nasopharyngeal swabs collected) than in breast milk (35.2%; 127 out of 361 women with all breast milk samples collected) and vaginal tract (24.5%; 92 out of 375 women with all vaginal tract samples collected).

Maternal GBS carriage during the 28 days following delivery is shown in Fig. 1(b). Overall, 31.6% of women (114 out of 361) were carriers at one or more time-points. Carriage at at least at one time-point was more common in the vaginal tract (23.5%; 88 out of 375) and breast milk (15.8%; 57 out of 361) than in NPS (1.1%; 4 out of 361). In the breast milk samples, carriage decreased from 7.7% (29 out of 377) at day 6 to 1.7% (6 out of 363) at day 28 (p < 0.001).

### Neonatal carriage of *S. aureus* and GBS

**Fig. 2:**
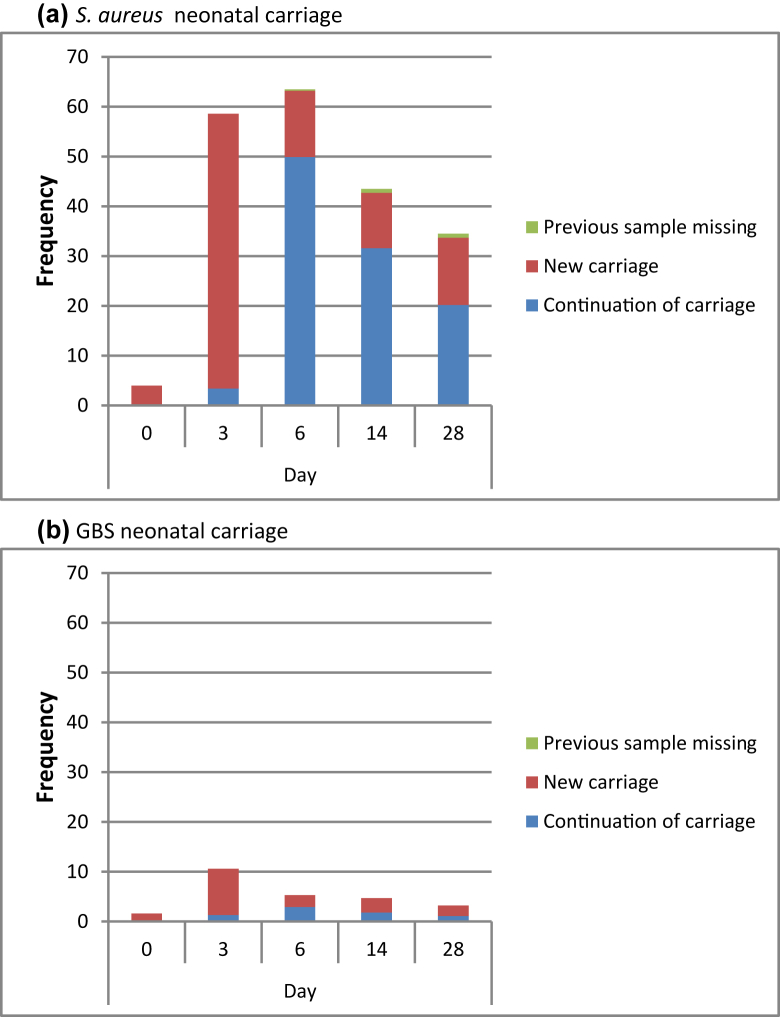
Frequency (%) of positive neonatal swabs for (a) *Staphylococcus aureus* and (b) Group B streptococcus at each time-point.

Most children (81.6%; 292 of the 358 children with all samples collected) were carriers of *S. aureus* at some time-point during the neonatal period. The prevalence of carriage increased rapidly from 4.0% at birth (15 out of 377) to a peak of 63.1% (238 out of 377) at day 6 (Fig. 1a) and decreased to 35.6% at day 28 (128 out of 360). The greatest number of new episodes of carriage occurred on day 3 (Fig. 2a).

Neonatal carriage of GBS was lower than that of *S. aureus*, but still relatively common (12.0%; 43 out of 358). New infections occurred throughout the neonatal period (Fig. 2b), although the number varied with time. The largest number occurred at day 3, coinciding with the peak prevalence (10.3%; 39 out of 377). One in 20 children (18 out of 366) were still carriers at day 14 (Fig. 1b).

### Association between maternal and neonatal colonization

Neonatal carriage of *S. aureus* at day 6 was associated with the presence of bacteria in the breast milk (adjusted OR 2.54; 95% CI 1.45–4.45), the mother's vaginal tract (aOR 2.55; 95% CI 1.32–4.92) and the mother's nasopharynx (aOR 2.49; 95% CI 1.56–3.97) (Table 2). Carriage of GBS was associated with the presence of bacteria in breast milk (aOR 3.75; 95% CI 1.32–10.65) and vaginal tract (aOR 3.42; 95% CI 1.27–9.22) (Table 2).

**Table 2:** Prevalence of *Staphylococcus aureus* and Group B streptococcus in newborns at day 6 by maternal bacterial carriage status

Site	Bacterial carriage		Neonatal carriage % (*n*)	aOR^a^ (95% CI)	p-value
***S. aureus***					
Vaginal swab	*S. aureus*	No	60.3 (188/312)		
		Yes	76.9 (50/65)	2.55 (1.32–4.92)	0.005
	GBS	No	62.2 (196/315)		
		Yes	67.7 (42/62)	1.28 (0.71–2.33)	0.414
NPS	*S. aureus*	No	54.8 (125/228)		
		Yes	75.8 (113/149)	2.49 (1.56–3.97)	<0.001
	GBS	No	62.9 (236/375)		
		Yes	100.0 (2/2)	—	—
Breast milk	*S. aureus*	No	58.4 (167/286)		
		Yes	78.0 (71/91)	2.54 (1.45–4.45)	0.001
	GBS	No	65.3 (216/331)		
		Yes	47.8 (22/46)	0.45 (0.23–0.85)	0.015
**GBS**					
Vaginal swab	*S. aureus*	No	4.5 (14/312)		
		Yes	9.2 (6/65)	2.69 (0.93–7.34)	0.069
	GBS	No	4.1 (13/315)		
		Yes	11.3 (7/62)	3.42 (1.27–9.22)	0.015
NPS	*S. aureus*	No	5.7 (13/228)		
		Yes	4.7 (7/149)	0.80 (0.31–2.09)	0.655
	GBS	No	5.3 (20/375)		
		Yes	0 (0/2)	NA	—
Breast milk	*S. aureus*	No	5.9 (17/286)		
		Yes	3.3 (3/91)	0.53 (0.15–1.86)	0.319
	GBS	No	4.2 (14/331)		
		Yes	13.0 (6/46)	3.75 (1.32–10.65)	0.013

Abbreviations: aOR, adjusted OR; GBS, Group B streptococcus; NPS, nasopharyngeal swab.

a OR adjusted by mother's age, ethnicity, mode of delivery and season.

Overall, 21% (95% CI 13–29) of neonatal *S. aureus* carriage and 31% (95% CI 2–51) GBS carriage was attributable to maternal colonization.

### Other risk factors for neonatal colonization

The prevalence of *S. aureus* carriage at day 6 varied between the different ethnic groups (Table 3). The prevalence of GBS carriage at day 6 was lower among neonates whose mothers were >30 years of age and those who had more than two children living with them in the household (Table 4).

**Table 3:** Epidemiological and demographic characteristics as potential risk factors for *Staphylococcus aureus* nasopharyngeal carriage in newborns at day 6

Variable	Categories	*n*	Carriage with *S. aureus* at day 6		
			% (*n*)	OR (95% CI)	p-value
*Delivery characteristics*					
Season	Dry (November–May)	251	60.6 (152)		
	Rainy	126	68.3 (86)	1.40 (0.89–2.20)	0.174
Day of the week	Weekend	52	59.6 (31)		
	Week day	325	63.7 (207)	1.19 (0.65–2.16)	0.643
Time of the day	Not working hours	121	63.3 (162)		
	Working hours (8 am–4 pm)	256	62.8 (76)	1.02 (1.32–2.18)	0.931
Low birthweight	No	348	63.5 (221)		
	Yes	29	58.6 (17)	0.81 (0.38–1.76)	0.689
Gender	Female	177	62.1 (110)		
	Male	200	64.0 (128)	1.09 (0.72–1.65)	0.749
Apgar score (at birth)	1–6	4	50.0 (2)		
	7–10	372	63.2 (235)	1.72 (0.24–12.32)	0.628
Hours from membrane rupture to birth	<1 hour	136	61.8 (84)		
	>1 hour	53	60.4 (32)	0.94 (0.49–1.81)	0.869
*Maternal characteristics*					
Maternal age	18–19 years	36	69.4 (25)	Baseline	0.631
	20–29 years	247	61.5 (152)	0.70 (0.33–1.50)	
	≥30 years	94	64.9 (61)	0.80 (0.36–1.86)	
Ethnic group	Mandinka	169	66.3 (113)	Baseline	0.029
	Wollof	43	72.1 (31)	1.31 (0.63–2.75)	
	Jola	52	65.4 (34)	0.96 (0.50–1.85)	
	Fula	57	61.4 (35)	0.81 (0.43–1.51)	
	Other	51	43.1 (22)	0.39 (0.20–0.73)	
Formal education	<1 year	187	63.1 (118)		
	≥1 year	181	62.4 (113)	0.97 (0.64–1.48)	0.914
*Household characteristics*					
Mother baths the newborn	No	161	67.1 (108)		
	Yes	203	60.1 (122)	0.74 (0.48–1.14)	0.171
Mother cooks with child on the back	No	334	62.3 (208)		
	Yes	31	61.3 (19)	0.96 (0.45–1.84)	0.914
Children <5 years sleeping with newborn	No	203	60.6 (123)		
	Yes	168	64.9 (109)	1.20 (0.79–1.84)	0.396
People in the household	2–4	192	62.5 (120)		
	>4	181	63.0 (114)	1.02 (0.67–1.55)	0.923
Three quantiles of Socio Economical Status score	1, poorest	109	57.8 (63)	Baseline	0.308
	2	117	67.5 (79)	1.52 (0.88–2.61)	
	3, least poor	101	64.4 (65)	1.32 (0.76–2.30)	
People smoke	No	343	63.8 (219)		
	Yes	31	51.6 (16)	0.60 (0.29–1.26)	0.181
Use of camphor	No	269	63.6 (171)		
	Yes	105	61.0 (64)	0.89 (0.56–1.42)	0.638

**Table 4:** Epidemiological and demographic characteristics as potential risk factors for group B streptococcus nasopharyngeal carriage in newborns at day 6

Variable	Categories	*N*	Carriage with group B streptococcus at day 6		
			% (*n*)	OR (95% CI)	p-value
*Delivery characteristics*					
Season	Dry (November–May)	251	5.6 (14)		
	Rainy	126	4.8 (6)	0.85 (0.32–2.26)	0.813
Day of the week	Weekend	52	3.8 (2)		
	Week day	325	5.5 (18)	1.47 (0.33–6.51)	1.000
Time of the day	Not working hours	121	5.1 (13)		
	Working hours (8 am–4 pm)	256	5.8 (7)	1.15 (0.45–2.95)	0.807
Low birthweight	No	348	5.7 (20)		
	Yes	29	0	NA	NA
Gender	Female	177	3.9 (7)		
	Male	200	6.5 (13)	1.71 (0.67–4.38)	0.265
Apgar score (at birth)	1–6	4	0		
	7–10	372	5.4 (20)	NA	NA
Hours from membrane rupture to birth	<1 hour	136	4.4 (6)		
	≥1 hour	53	9.4 (5)	2.26 (0.66–7.74)	0.297
*Maternal characteristics*					
Maternal age	18–19 years	36	2.8 (1)	Baseline	0.044
	20–29 years	247	7.3 (18)	2.75 (0.36–21.26)	
	≥30 years	94	1.1 (1)	0.38 (0.02–6.18)	
Ethnic group	Mandinka	169	6.5 (11)	Baseline	0.760
	Wollof	43	2.3 (1)	0.34 (0.04–2.72)	
	Jola	52	7.7 (4)	1.20 (0.36–3.93)	
	Fula	57	3.5 (2)	0.53 (0.11–2.43)	
	Other	51	3.9 (2)	0.59 (0.13–2.74)	
Formal education	<1 year	187	5.3 (10)		
	≥1 year	181	5.5 (10)	1.04 (0.42–2.55)	1
*Household characteristics*					
Mother baths the newborn	No	161	6.8 (11)		
	Yes	203	4.4 (9)	0.63 (0.26–1.57)	0.322
Mother cooks with child on the back	No	334	5.4 (18)		
	Yes	31	3.2 (1)	0.59 (0.08–4.45)	0.608
Children <5y sleeping with newborn	No	203	5.9 (12)		
	Yes	168	4.8 (8)	0.80 (0.32–1.99)	0.626
People in the household	2–4	192	5.7 (11)		
	>4	181	5.0 (9)	0.86 (0.35–2.13)	0.746
3 quantiles of Socio Economical Status score	1, poorest	109	4.6 (5)	Baseline	0.558
	2	117	5.1 (6)	1.12 (0.33–3.80)	
	3, least poor	101	7.9 (8)	1.79 (0.57–5.66)	
People smoke	No	343	5.0 (17)		
	Yes	31	9.7 (3)	2.05 (0.57–7.44)	0.273
Use of camphor	No	269	6.7 (18)		
	Yes	105	1.9 (2)	0.27 (0.06–1.19)	0.083

## Discussion

This study has shown that carriage of *S. aureus* and GBS is common among Gambian women and their newborns, and that maternal colonization is an important risk factor for neonatal colonization. Neonates were more likely to be colonized with *S. aureus* and GBS if the bacteria were present in the mother's vaginal tract, breast milk or nasopharynx (only *S. aureus* for the last of these). Combined, these sites explained 21% of *S. aureus* neonatal colonization and 31% of GBS neonatal colonization at day 6.

In keeping with our results, in Gabon, *S. aureus* maternal carriage was associated with neonatal carriage [7], though only 5.6% of neonatal colonization was attributable to maternal transmission. The difference between this estimate and our estimate could be explained by the fact that investigators in Gabon did not collect vaginal swabs and the fact that they collected samples from babies after the neonatal period, when other sources of infection may have played a greater role. The fraction of neonatal carriage explained by maternal carriage in our study is probably also underestimated because important sources of maternal transmission were not sampled (i.e. oropharyngeal and nasal swabs for *S. aureus* and rectal swabs for GBS); and additional sources of neonatal carriage were not evaluated (i.e. skin, umbilicus, rectum and ears).

Maternal vaginal GBS carriage and breast milk carriage were common and persisted throughout the neonatal period. GBS carriage in breast milk has the potential to increase the risk of late-onset neonatal sepsis [17], and this may explain why the incidence of GBS disease remains high in sub-Saharan Africa after the early neonatal period [18]. Our results suggest that vaginal screening for GBS carriage would not suffice to identify children at high risk of invasive GBS disease [9], [10], [19] as 18% of women who had a negative swab at birth were carriers of GBS at some point during the neonatal period. However, some of these women may have been colonized at birth because we used sub-optimal methods for investigating for GBS colonization (i.e. vaginal rather than rectovaginal).

Our analysis has several limitations. First, as previously mentioned, we did not collect oropharyngeal, nasal or rectal swabs from the mother or skin, umbilical, rectal and ear swabs from the babies. The fact that we did not collect these swabs is of particular importance because it is likely that we underestimated the prevalence of maternal and neonatal colonization of both *S. aureus* and GBS; and diluted the fraction of neonatal carriage explained by maternal carriage. Second, carriage prevalence may have been underestimated because only healthy mothers and their babies were recruited into the study, and women and babies who participated in the trial received better health care. Third, swabs were not collected from siblings and other close contacts, so it was not possible to compare maternal transmission with that from other sources. Fourth, we cannot exclude the possibility that the observed association between neonatal colonization at day 6 and maternal colonization is explained, at least in part, by transmission from the neonate to the mother as was observed in the Gabon study [7]. Lastly, the study was designed to assess the impact of maternal carriage on neonatal carriage, it was not designed to assess its impact on neonatal disease.

Due to the high burden of maternal colonization and the role of the mothers in neonatal colonization, Gambian newborns are rapidly colonized by *S. aureus* and GBS over the first month of life and therefore are at high risk for neonatal sepsis. Interventions that target pregnant women in sub-Saharan Africa are likely to reduce neonatal carriage and associated disease. On account of the high burden of maternal carriage in the region, it may be reasonable to assume that most children in sub-Saharan Africa are at high risk of neonatal sepsis. Interventions that target maternal colonization (e.g. maternal antibiotics or maternal vaccination) are expected to reduce neonatal colonization and associated sepsis. However, other sources of neonatal colonization should also be considered. For prevention strategies that involve the use of wide-spectrum prophylactic antibiotics, continuous monitoring of the potentially negative effect of the intervention on antibiotic resistance will need to be in place.

## Transparency declaration

All authors declare that there are no competing interests.

## Authors' contributions

AR conceived the study and designed the study, drafted the protocol and wrote the initial manuscript. CB gave support to the statistical analysis and significantly contributed to the manuscript. UDA contributed significantly to the final version of the design, protocol and manuscript. AB, BC and CO developed and adapted the laboratory and field work and made contributions to the development of the manuscript. KL and PW led the data management and the statistical analysis and contributed to the manuscript. All authors read and approved the final manuscript.

## Financial disclosure

The MRC Unit in The Gambia receives core funding from the MRC UK. This trial was jointly funded by the UK MRC and the UK Department for International Development (DFID) under the MRC/DFID Concordat agreement (reference number MR/J010391/1) and is also part of the EDCTP2 programme supported by the European Union.
